# Monthly Summary

**Published:** 1877-04

**Authors:** 


### MONTHLY SUMMARY.

---

*Partial Exsection of both Upper Jaws for Enchondroma.*—Dr. Porter reported to the Boston Society for Medical Improvement (*Boston Med. and Surg. Journ.*,) the following interesting case of enchondroma of the upper jaw. The patient, a gentleman aged fifty-one, of excellent health, first noticed, nine years ago, a congested condition around the two central incisors of the upper jaw. These were "pivot" teeth, inserted twenty years before, and as they often became loose, he had been in the habit of replacing the pivots by the ends of matches. There finally resulted a necrosis of the alveolar process around these teeth, which gradually exoliated. Soon a defined tumour commenced in the above locality, which has been growing for three years. It has been for the most part painless, but has doubled in size within six months. The patient's family history shows no record of tumors.

The tumor now involves the upper alveolar arch nearly symmetrically on both sides, from the first molar tooth on one side, to the corresponding tooth on the opposite side, extending nearly up to the infra-orbital foramina, pushing upwards and forwards the nose and upper lip, producing a very considerable deformity.

The operation for the removal of the growth was performed with ether. A Y-shaped incision was first made, the long arm of the Y cutting through the median line of the lip and the short arms extending one into each nostril. The coronary artery was controlled by a deep temporary suture in each flap. The soft parts on both sides were then dissected up as high as the level of the infra-orbital foramen, and backwards to the tuberosity of the superior maxilla. The hemorrhage was readily controlled by sponges stuffed into the wound. The second molar tooth on each side was then drawn, the mucous membrane covering the hard palate cut transversely, and the alveolar process and hard palate sawed through from side to side, and upwards quite into the antra. The anterior wall of the antrum and the nasal process on each side were cut through by a few quick strokes with mallet and chisel, the septum nasi severed at the union with the palate process, and the tumour easily depressed and removed entire. A number of large vessels required ligature, though much less blood was lost than would be supposed from the extensive laceration of the soft parts. The wound was brought together by sutures. The pulse remained good throughout.

The tumour, examined by Dr. R. H. Fitz proved to be enchondroma. The patient made a rapid convalescence, and returned home on the eighth day after the operation.

In four months from the first operation, he noticed a return of the growth on each side of the nose, and when seen two months later, the tumour of the right side was about the size of an English walnut, involving the nasal process of the superior maxilla; that of the left side was similarly situated, but less in extent and size, being as large as a chestnut.

Before the operation for the removal of the tumour was commenced, the patient was etherized and tracheotomy was done. Sponges were then introduced into the pharnyx to prevent bleeding into the throat. As in the first operation, a Y-shaped incision was made through the middle of the lip upwards into each nostril. The cheeks were freely dissected up on the right side as high as the inner commissure of the eyelids, and on the left to a level with the floor of the orbit The bony attachments were then cut with chisel and mallet, the whole nasal process being removed on the right side, and nearly the whole on the left. The wound was closed as before. The tracheotomy-tube was left in for a few hours, until all bleeding had ceased, when it was removed and the wound closed. The patient rallied well from the operation, and made a more rapid recovery than on the first occasion, returning home on the seventh day. The tumours, examined by Dr. Fitz, were like the first, but richer in cells.

Dr. Porter said he wished to call attention to but one practical point in the first operation, and that was the small extent of the incision. The Y-shape of the cut through the median line of the lip, into each nostril, made the circumference of the nostril available and gave an abundance of room for the operation ; the resulting cicatrix was very small and entirely covered by the mustache. This method of incision, moreover, results in no paralysis of any of the facial muscles. In the second operation, tracheotomy beforehand did away with the constant sponging of the throat consequent upon the character of the operation, and also with the danger from blood in the trachea.

Enchondroma of the jaws is a rare disease, and particularly so that of the upper jaw. In the *Dublin Quarterly Journal of Medical Science* for November, 1857, Dr. Oscar Heyfelder, of Munich, reports only eight cases of enchondroma out of four hundred and fifty cases of diseases of the superior maxilla. Dr. Porter can find but few reported cases of partial or total exsection of both upper jaws. Partial resection of both bones was done in 1824 by Roger, afterwards by Liston and Dupuytren ; but the first total removal of both upper jaws was done by Dr. J. F. Heyfelder, of Munich, who did it three times in 1844, and afterwards in 1850 and 1852.

---

*Ununited Fracture of the Forearm, with Deficiency of the Ulna, treated successfully by Excision and the Wire Suture.*—Mr. Thomas Annandale, Surgeon to the Edinburgh Royal Infirmary, reports (*British Medical Journal*, Jan. 9, 1875) the following interesting case :—

R. K., aged 29, was admitted into my wards on June 24th, 1873, suffering from an ununited fracture of the bones of the forearm. About six months before his admission, his forearm had been severly injured by machinery. Both bones were fractured, and a large lacerated wound was caused by the accident. He was taken to a provincial hospital, and carefully treated for several months. About three months after the accident, a large piece of bone (a portion of the ulna) gradually loosened, and was removed. Three weeks after this the wound was healed, but the bones had not united properly.

When the arm was examined, a large cicatrix was noticed over the middle third of the bones of the forearm ; it was adherent to the ulna for a short distance, but was otherwise free. Both bones were movable at the junction of their middle and lower thirds, but the radius less so than the ulna.

The ulna was not only quite ununited, but was deficient for about one inch at the seat of fracture, the result, no doubt, of the necrosis which had followed the injury. The fractured ends of the ulna were displaced towards, and adherent to, the radius. Pronation and supination could not be performed, and the arm was also weak, and, in consequence, useless.

On June 27th, I performed the following operation, with the hope of making the arm more useful. An incision, about three inches long, was made over the dorsal aspect of the ulna, so as to expose the fractured portion of this bone. It was then found that the fractured ends were rounded off and atrophied, and united to one another and to the radius by some strong fibrous texture. These ends were also displaced inwards, and there was fully an interval of an inch between them, owing to the deficiency of the bone.

About a quarter of an inch was now sawn off the ends of the ulna, and, as it was quite evident that these ends could not be brought together, a second incision was made over the dorsal aspect of the radius, and a portion of this bone, including the partially united part, was also sawn off. By thus shortening the radius to a sufficient extent, the ends of the ulna were allowed to meet, the adhesions connecting them to the former bone having been divided. The ends of both bones were then drilled and secured with strong silver wire. The edges of the wounds being brought together with a few carbolized silk sutures, antiseptic muslin was applied in the usual way, and the arm adjusted on a splint. On the 3d of July, it is noted that the patient has progressed favourably since the operation, and the wound is healing well. On the 8th of July, the wire through the ends of the ulna, being a little loose, was twisted more firmly. The patient's progress continues good.

On the 3d of August, the wounds were quite superficial, and the wire through the ends of the radius, being quite loose, was removed. On the 13th of August, the wire was removed from the ulna; and on the 29th the patient left the hospital, the wounds being almost healed.

Six weeks after this he returned to show himself, when it was found that the bones were firmly united. The forearm, to a limited extent, could be pronated and supinated; but these and the other movements of the arm were steadily improving, and the limb could already be used in many ways, its strength being improved since the operation.

*Remarks.*—For the successful treatment of this case, it was necessary to overcome two principal obstacles. These were: (1) the deficiency of the ulna; (2) the displacement inwards of the ends of the ulna, and their adhesion to the radius. In addition, the large cicatrix forming the chief covering of soft parts over the injured bones made operative interference more difficult than if these coverings had been sound. The first of these obstacles was successfully overcome by removing a portion of the radius, so as to allow the ends of both bones to be brought together. The removal of this portion of bone, by diminishing the amount of the osseous element of the forearm, also permited the contraction of the wounds in the soft parts to take place satisfactorily. The second obstacle was successfully combatted by dividing the adhesions, drilling the ends of both bones, and securing them with strong wire.

This method of securing the fractured ends would, I believe, prove very valuable in many cases of recent compound fracture of the bones of the forearm. It is a most efficient means of preventing their inward displacement, and therefore assists much in preventing also the union of the radius and the ulna to one another, a condition not easy to overcome in this class of injury. The wire which I employ in this and other operations of the kind is silver, of the thickness of that usually employed to secure the corks of soda-water bottles. The instrument used for drilling the bone is a joiner's small pricker. Having tried more complicated instruments for this purpose, I have now a decided preference for the more simple tool, which I always find to be most efficient.—*Monthly Abstract.*

---

*Venous Pulse an Habitual Symptom of the Physiological Action of Chloroform.*—(*Bulletin de l' Academie de Medicine de Belique. Med. Record*, Jan. 20, 1877.)—Prof. L. Noel at Louvain first observed this phenomenon. It appears always at the same period of the anæsthesia, that is, during the period of awaking, The internal jugular, the subclavian, in more than half the cases, the external jugulars, and sometimes even the facial veins are then the seat of isochronous pulsations with the radial pulse. They appear very marked to the eye, but give only a slight sensation to the finger. They continue about half an hour, diminishing, gradually, in intensity. During all this time the heart's action and the respirations present no particular modification. The professor thinks this indicates a profound perturbation of the functions of the heart, and proves that the organism is still under the influence of the anæsthetic. He says that it is due, possibly, to an incomplete closure of the right auriculo-ventricular valve ; on the other hand, it is not unlikely that the poison causes an engorgement of the venæ cavæ and the right ventricule, which prevents the escape of the contents of the auricle into the latter. Whatever may be the cause, it is important for surgeons to watch their patients while they are recovering from an anæsthetic.—*Detroit Medical Journal.*

